# Distributed Support Vector Ordinal Regression over Networks

**DOI:** 10.3390/e24111567

**Published:** 2022-10-31

**Authors:** Huan Liu, Jiankai Tu, Chunguang Li

**Affiliations:** College of Information Science and Electronic Engineering, Zhejiang University, Hangzhou 310027, China

**Keywords:** ordinal regression, support vector machine, support vector ordinal regression, distributed algorithm, subgradient method

## Abstract

Ordinal regression methods are widely used to predict the ordered labels of data, among which support vector ordinal regression (SVOR) methods are popular because of their good generalization. In many realistic circumstances, data are collected by a distributed network. In order to protect privacy or due to some practical constraints, data cannot be transmitted to a center for processing. However, as far as we know, existing SVOR methods are all centralized. In the above situations, centralized methods are inapplicable, and distributed methods are more suitable choices. In this paper, we propose a distributed SVOR (dSVOR) algorithm. First, we formulate a constrained optimization problem for SVOR in distributed circumstances. Since there are some difficulties in solving the problem with classical methods, we used the random approximation method and the hinge loss function to transform the problem into a convex optimization problem with constraints. Then, we propose subgradient-based algorithm dSVOR to solve it. To illustrate the effectiveness, we theoretically analyze the consensus and convergence of the proposed method, and conduct experiments on both synthetic data and a real-world example. The experimental results show that the proposed dSVOR could achieve close performance to that of the corresponding centralized method, which needs all the data to be collected together.

## 1. Introduction

Many real-world data labels have natural orders that are usually called ordinal labels. For example, fault severity in industrial processes is usually divided into {*harmless, slight, medium, severe*}. Ordinal regression, which aims at predicting ordinal labels for given patterns, has attracted a great deal of research in many fields, such as disease severity assessment [1], satisfaction evaluation [2], wind-speed prediction [3], age estimation [4], credit-rating prediction [5], and fault severity diagnosis [6]. Although classical classification and regression methods can be applied to the ordinal regression problem [7,8], they require additional prior information about the distances between labels. Otherwise, they often perform unsatisfactorily since they cannot fully use ordering information [9,10].

To tackle the aforementioned problems of classical classification and regression methods, many ordinal regression methods were proposed [10]. Among them, the most popular type of approaches are threshold models, which assume that a continuous latent variable underlies the ordinal response [10]. In threshold models, the order of the labels is represented by a set of ordered thresholds. These ordered thresholds define a series of intervals, and the data label depends on the interval the corresponding latent variable falls into. Among the threshold models, support vector ordinal regression (SVOR) [11,12] is widely used because of good generalization performance. A representative work is the support vector ordinal regression with implicit constraints (SVORIM) proposed in [11,12]. This determines each threshold by taking all the samples into consideration, where the threshold inequality constraints can be satisfied without explicit constraints.

Most of the existing ordinal regression methods have been developed in a centralized framework. However, in practice, data used for ordinal regression may be distributed in a network [13]. Each node of the network collects and stores part of the data, and it is not enough for a single node to train a model with good performance. For instance, in industrial processes, sensors are often used in factories to monitor the operating status of equipment and diagnose fault severity. Due to the rarity of faults, a single sensor can only collect very few data, and the faults encountered by each factory may also be different. To train a proper model, we need to use as many data as possible. However, in some realistic scenarios, it is difficult for data to be transmitted to a central node for various reasons [13]. For example, factories may not want to leak data regarding their equipment in order to protect privacy. Moreover, if the data are collected by image sensors or video sensors, it may be difficult for a single machine to store and process such a large amount of data. In such situations, centralized methods are inapplicable, and distributed methods are more suitable choices.

In this paper, we propose a distributed support vector ordinal regression algorithm based on the SVORIM method to deal with more complex nonlinear problems in distributed ordinal regression. First, we formulate a constrained optimization problem for SVORIM in the distributed scenarios. Classical methods usually solve the problem by transforming it into the dual problem. In distributed circumstances where the original data cannot be transmitted to others, it is difficult for classical methods to calculate the kernel function values and optimize the dual variables because they require data from different nodes. Thus, we adopted a random approximation method and the hinge loss function to transform the optimization problem to overcome the above difficulties. Increasing the number of random approximation dimensions can improve the approximation accuracy, but brings redundancy. In order to find an appropriate number of approximation dimensions, we further added a sparse regularization term of the approximation dimension number to the objective function. Through the above steps, we transformed the original problem into a convex optimization problem with consensus constraints. Then, to solve the problem, we propose a subgradient-based algorithm called distributed SVOR (dSVOR) where each node only uses its own data and the parameter estimates exchanged from its neighbors. To verify the effectiveness of dSVOR, we theoretically analyze its consensus and convergence, and conducted some experiments on synthetic data and a real-world example. The experimental results show that the proposed distributed algorithm under additional constraints could achieve close performance to that of the corresponding centralized method, which needs all the data to be collected to a central node.

The main contributions of this paper are summarized as follows.

1. Existing work on distributed ordinal regression [14] uses a linear model; therefore, it cannot deal with the problems of linearly inseparable data. We extended the SVOR method to distributed scenarios to solve distributed ordinal regression problems with linearly inseparable data.
2. We developed a decentralized implementation of SVOR, and propose a dSVOR algorithm. In the proposed algorithm, the kernel feature map is approximated by random feature maps to avoid transmitting the original data, and sparse regularization is added to avoid excessively high approximation dimensions.
3. The consensus and convergence of the proposed algorithm are theoretically analyzed.

The rest of this paper is organized as follows. In Section 2, we introduce related works. The ordinal regression problem and the SVORIM method are introduced in Section 3 as preliminary knowledge. In Section 4, we formulate the distributed support vector ordinal regression problem, propose the dSVOR algorithm, and perform theoretical analysis of the proposed algorithm. Experiments were conducted to evaluate the effectiveness of the proposed algorithm and they are presented in Section 5. Lastly, in Section 6, we draw some conclusions.

## 2. Related Works

**Ordinal Regression Methods.** Many ordinal regression methods have been proposed to solve ordinal regression problems. The ordered logit model [15,16] makes assumptions about the distribution of the prediction error of the latent variable, and uses the cumulative distribution function to build the label cumulative probability function. The support vector ordinal regression (SVOR) [11,12] maximizes margins between two adjacent labels. Variants of SVOR with nonparallel hyperplanes were discussed in [17,18]. There are also ordinal regression methods that solve ordinal regression problems by solving a series of binary classification subproblems. In [4,19], extended labels were extracted from the original ordinal labels to learn a binary classifier (such as support vector machine [19] or logistic regression [4]); then, a ranking rule was constructed from the binary classifier to predict ordinal labels. In [20], the authors used the stick-breaking process to construct a series of binary classification subproblems to guarantee that the cumulative probabilities were monotonically decreasing. However, the above ordinal regression methods are all centralized and are infeasible in distributed scenarios.

**Distributed methods.** Distributed methods were extensively studied in many fields, such as distributed estimation [21,22], distributed optimization [23,24], distributed clustering [25], distributed Kalman filter [26], and distributed anomaly detection [27]. However, as far as we know, there are few works investigating distributed ordinal regression [14]. In [14], the authors proposed a distributed generalized ordered logit model, which is a linear model and therefore cannot handle complex problems.

## 3. Preliminaries

### 3.1. Ordinal Regression Problem

The classification problem aims at classifying the *K*-dimensional input vector $x\in X\subseteq R^{K}$ into one of *Q* discrete categories $y\in Y=\{C_{1},C_{2},\ldots ,C_{Q}\}$. The ordinal regression problem is a type of classification problem in which the data labels have a natural order $C_{1}≺C_{2}≺\cdots ≺C_{Q}$, where ≺ is an order relation [10]. The purpose of ordinal regression is to find a mapping function f:X→Y to predict the ordinal labels for new patterns given a training set of *N* samples $D=\{\left(x_{i},y_{i}\right),i=1,\ldots ,N\}$.

### 3.2. Support Vector Ordinal Regression with Implicit Constraints

Let ϕ(x) denote the feature vector in a high-dimensional reproducing kernel Hilbert space (RKHS) of input vector x. The inner product in the RKHS is defined by the reproducing kernel function: $K\left(x,x^{\prime }\right)=\phi \left(x\right)\cdot \phi \left(x^{\prime }\right)$.

Support vector machines construct a discriminant hyperplane in the RKHS by maximizing the distance between support vectors and the discriminant hyperplane. The discriminant hyperplane is defined by an optimal direction w and a single optimal threshold *b*. It divides the feature space into two regions for two classes.

The support vector ordinal regression constructs Q−1 parallel discriminant hyperplanes for *Q* ordinal labels where these hyperplanes are defined by optimal direction w and Q−1 thresholds ${\left\{b_{q}\right\}}_{q=1,\ldots ,Q-1}$. The ordinal information in the labels is represented by threshold inequalities $b_{1}\leq b_{2}\leq \cdots \leq b_{Q-1}$. For convenience, vector $b={\left[b_{1}\;b_{1}\cdots b_{Q-1}\right]}^{T}$ was used to denote these thresholds.

In [11,12], the SVORIM method determined a threshold $b_{q}$ by utilizing the samples of all the labels. For threshold $b_{q}$, each sample belonging to $C_{p},\forall p\leq q$ should have a function value less than $b_{q}-1$; otherwise, $\xi_{pi}^{q}=w\cdot \phi \left(x_{i}^{p}\right)-\left(b_{q}-1\right)$ is the empirical error of $x_{i}^{p}$ for $b_{q}$. Similarly, each sample belonging to $C_{p},\forall p>q$ should have a function value greater than $b_{q}+1$; otherwise, $\xi_{pi}^{*q}=\left(b_{q}+1\right)-w\cdot \phi \left(x_{i}^{p}\right)$ is the empirical error of $x_{i}^{p}$ for $b_{q}$.

As proved in [11,12], this approach has the property that the threshold inequalities can be automatically satisfied after convergence without explicitly including the corresponding constraints. This method is called support vector ordinal regression with implicit constraints and is formulated as follows:

(1) $$\begin{array}{cc} \min_{w,b,\xi ,\xi^{*}}\; & \frac{1}{2}{∥w∥}^{2}+C\sum_{q=1}^{Q-1}\sum_{p=1}^{q}\sum_{i=1}^{N^{p}}\xi_{pi}^{q}+C\sum_{q=1}^{Q-1}\sum_{p=q+1}^{Q}\sum_{i=1}^{N^{p}}\xi_{pi}^{*q}\\ s.t.\;\; & w\cdot \phi \left(x_{i}^{p}\right)-b_{q}\leq -1+\xi_{pi}^{q},\;\xi_{pi}^{q}\geq 0,\;\forall i,q\;\mathrm{and}\;p=1,\ldots ,q\\ \; & w\cdot \phi \left(x_{i}^{p}\right)-b_{q}\geq +1-\xi_{pi}^{*q},\;\xi_{pi}^{*q}\geq 0,\;\forall i,q\;\mathrm{and}\;p=q+1,\ldots ,Q, \end{array}$$

where *C* is a predefined positive constant. The above problem can be solved by solving the dual problem, which can be derived with standard Lagrangian techniques. Let $\beta_{pi}^{q}\geq 0,\gamma_{pi}^{q}\geq 0,\beta_{pi}^{*q}\geq 0$, and $\gamma_{pi}^{*q}\geq 0$ be the Lagrangian multipliers for the constraints in the above equation. The dual problem is the following maximization problem [11,12].

(2) $$\begin{array}{cc} \max_{\beta ,\beta^{*}}\; & -\frac{1}{2}\sum_{p,i}\sum_{p^{\prime },i^{\prime }}\left(\sum_{q=1}^{p-1}\beta_{pi}^{*q}-\sum_{q=p}^{Q-1}\beta_{pi}^{q}\right)\left(\sum_{q=1}^{p^{\prime }-1}\beta_{p^{\prime }i^{\prime }}^{*q}-\sum_{q=p^{\prime }}^{Q-1}\beta_{p^{\prime }i^{\prime }}^{q}\right)K\left(x_{i}^{p},x_{i^{\prime }}^{p^{\prime }}\right)\\ \; & \;+\sum_{p,i}\left(\sum_{q=1}^{p-1}\beta_{pi}^{*q}+\sum_{q=p}^{Q-1}\beta_{pi}^{q}\right)\\ s.t.\; & \sum_{p=1}^{q}\sum_{i=1}^{N^{p}}\beta_{pi}^{q}=\sum_{p=q+1}^{Q}\sum_{i=1}^{N^{p}}\beta_{pi}^{*q},\;\forall q\\ \; & 0\leq \beta_{pi}^{q}\leq C,\;\forall i,q\;\mathrm{and}\;p\leq q\\ \; & 0\leq \beta_{pi}^{*q}\leq C,\;\forall i,q\;\mathrm{and}\;p>q. \end{array}$$

For a new pattern x, SVORIM calculates the function value w·ϕ(x) and then decides its category according to the interval the function value falls into, where the intervals are defined by thresholds ${\left\{b_{q}\right\}}_{q=1,\ldots ,Q-1}$.

## 4. Distributed Support Vector Ordinal Regression Algorithm

### 4.1. Network and Data Model

In this paper, we consider a network consisting of *M* nodes. We could use a graph G=(M,E) to represent this network. It consisted of a set of nodes M={1,2,…,M} and a set of edges E. Each edge (m,n)∈E connected a pair of distinct nodes. We used $N_{m}=\left\{n|\left(m,n\right)\in E\right\}$ to represent the set of neighbors of node m∈M.

Data used for ordinal regression are distributedly collected and stored by the *M* nodes of this network. The *i*-th sample of node *m* is represented as $\left(x_{m,i},y_{m,i}\right)$, where $x_{m,i}\in X$ and $y_{m,i}\in Y$. More specifically, at node *m*, the total number of samples is $N_{m}$, the number of samples that belong to $C_{q}$ is $N_{m}^{q}$, and the *i*-th sample of $C_{q}$ is denoted as $\left(x_{m,i}^{q},y_{m,i}^{q}\right)$.

**Figure 1:**
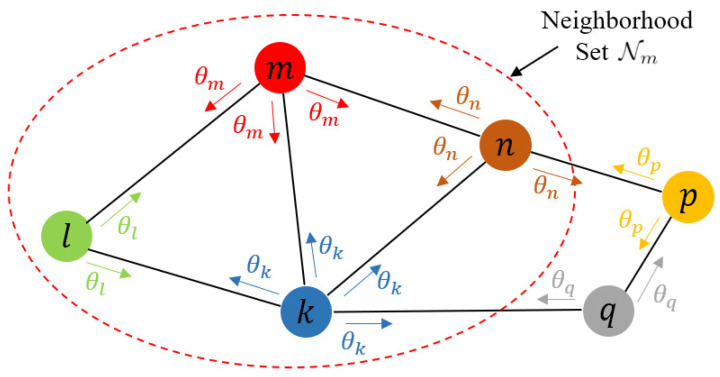
Schematic of a distributed network. Node *m* only transmits its parameters $\theta_{m}$ with nodes in $N_{m}$.

Figure 1 shows a schematic of a distributed network. In distributed networks, due to limited storage, computation and communication resources and the need for privacy protection, node *m* can only transmit some parameters $\theta_{m}$ instead of the original data to its neighbor nodes in $N_{m}$, and perform local computation using only its own data ${\left\{\left(x_{m,i},y_{m,i}\right)\right\}}_{1\leq i\leq N_{m}}$ and the parameters exchanged from its neighbors. Each node should eventually obtain a model consensus with that obtained by other nodes, and the performance of the model should be close to that of the model trained using all the data.

### 4.2. Problem Formulation

In centralized SVOR, the objective is to find an optimal direction w and a vector b. If the data from all the nodes of the distributed network can be collected together, then parameters θ={w,b} can be obtained by solving Problem (1).

In distributed situations, data are not allowed to be transmitted to a central node. Each node can only use its own data and some parameters from its neighbors. In this case, each node *m* has a local estimate $\theta_{m}$ of θ. With a connected network, we imposed constraints $\theta_{m}=\theta_{n},\forall \left(m,n\right)\in E$ to ensure the consensus of ${\left\{\theta_{m}\right\}}_{m=1,\ldots ,M}$. Then, the corresponding optimization problem in distributed scenarios can be written as follows:

(3) $$\begin{array}{cc} \min\;\; & \frac{1}{2}\sum_{m=1}^{M}{∥w_{m}∥}^{2}+C\sum_{m=1}^{M}\sum_{q=1}^{Q-1}\sum_{p=1}^{q}\sum_{i=1}^{N_{m}^{p}}\xi_{m,pi}^{q}+C\sum_{m=1}^{M}\sum_{q=1}^{Q-1}\sum_{p=q+1}^{Q}\sum_{i=1}^{N_{m}^{p}}\xi_{m,pi}^{*q}\\ s.t.\;\; & w_{m}\cdot \phi \left(x_{m,i}^{p}\right)-b_{m,q}\leq -1+\xi_{m,pi}^{q},\;\xi_{m,pi}^{q}\geq 0,\\ \; & \forall m,i,q\;\mathrm{and}\;p=1,\ldots ,q\\ \; & w_{m}\cdot \phi \left(x_{m,i}^{p}\right)-b_{m,q}\geq +1-\xi_{m,pi}^{*q},\;\xi_{m,pi}^{*q}\geq 0,\\ \; & \forall m,i,q\;\mathrm{and}\;p=q+1,\ldots ,Q\\ \; & w_{m}=w_{n},b_{m}=b_{n},\forall \left(m,n\right)\in E, \end{array}$$

where $\xi_{m,pi}^{q}$ is the empirical error of $x_{m,i}^{p}$ for $b_{m,q}$ when p=1,…,q and $\xi_{m,pi}^{*q}$ is the empirical error of $x_{m,i}^{p}$ for $b_{m,q}$ when p=q+1,…,Q. With the help of the consensus constraints, this problem is equivalent to Problem (1).

### 4.3. Problem Transformation

In classical solutions, a primal problem is solved by solving the corresponding dual problem. Applying such methods to Distributed Problem (3) is confronted with two major difficulties:

1. For nonlinear kernel functions, the dimension of the RKHS is unknown, and we can only calculate the inner product of $\phi (x_{m,i})$ and $\phi (x_{n,j})$ rather than them. Because the data are distributed in various nodes of the network, the kernel function $K(x_{m,i},x_{n,j})$ requiring data from different nodes is difficult to calculate without transmitting the original data.
2. The dual variables of samples should satisfy constraints in (2). In the distributed scenarios, the dual variables of the first constraint in (2) are usually from different nodes. Since each node is only allowed to exchange information with its neighbors, it is difficult to optimize these dual variables.

To overcome the first difficulty, we use a random approximate function [28] $z:R^{K}\to R^{D}$, where D>K, to map the data to a *D*-dimensional space instead of RKHS. In this study, for Gaussian kernel function

(4) $$K\left(x,x^{\prime }\right)=\exp\left(-\frac{∥x-x^{\prime }{∥}^{2}}{\sigma^{2}}\right),$$

we adopted $z\left(x\right)={\left[z_{\omega_{1}}\left(x\right),\ldots ,z_{\omega_{D}}\left(x\right)\right]}^{T}$, where each dimension $z_{\omega_{i}}\left(x\right)$ was

(5) $$z_{\omega_{i}}\left(x\right)=\sqrt{\frac{2}{D}}\cos\left(\omega_{i}^{T}x+\psi_{i}\right),$$

where $\psi_{i}$ is drawn uniformly from [0,2π], and $\omega_{i}$ is drawn from the Fourier transform of Gaussian kernel function

(6) $$p\left(\omega \right)={\left(2\pi \right)}^{-\frac{K}{2}}\exp\left(-\frac{\sigma^{2}{∥\omega ∥}^{2}}{2}\right).$$

As proved in [28], if dimensional number *D* is large enough, $z{\left(x\right)}^{T}z\left(x^{\prime }\right)$ can approximate $K(x,x^{\prime })$ well, and z(x) can approximate ϕ(x) well. According to Cover’s theorem [29], a complex pattern-classification problem nonlinearly cast in a high-dimensional space is more likely to be linearly separable than it is in a low-dimensional space. Therefore, to ensure good performance, we should set a relatively large *D*. For other shift-invariant kernels such as Laplacian and Cauchy, the authors in [28] provided corresponding finite-dimensional random approximate functions. For additive homogeneous kernels, such as Hellinger’s, $\chi^{2}$, intersection and Jensen-Shannon, the authors in [30] also provided efficient finite-dimensional approximate mapping functions. For a linear kernel function, random approximation is not necessary, so we defined z(x)=ϕ(x)=x.

With the random approximation, mapping function ϕ(x) in (3) is replaced by z(x). The calculation of z(x) only requires one data point from a single node instead of a pair of data from different nodes like the kernel function, so the first difficulty is solved.

After the random approximation is performed, the data are mapped into a *D*-dimensional feature space instead of the RKHS with unknown dimension. Thus, we could directly solve the primal problem instead of the dual problem, which automatically tackles the second difficulty.

With the use of hinge loss function L(x)=max(1−x,0) [31], the problem can be rewritten as follows:

(7) $$\begin{array}{cc} \min\;\; & \frac{1}{2}\sum_{m=1}^{M}{∥w_{m}∥}^{2}+C\sum_{m=1}^{M}\sum_{q=1}^{Q-1}\sum_{p=1}^{q}\sum_{i=1}^{N_{m}^{p}}L\left(b_{m,q}-w_{m}\cdot z\left(x_{m,i}^{p}\right)\right)\\ \; & +C\sum_{m=1}^{M}\sum_{q=1}^{Q-1}\sum_{p=q+1}^{Q}\sum_{i=1}^{N_{m}^{p}}L\left(w_{m}\cdot z\left(x_{m,i}^{p}\right)-b_{m,q}\right)\\ s.t.\;\; & w_{m}=w_{n},b_{m}=b_{n},\forall \left(m,n\right)\in E. \end{array}$$

### 4.4. Sparse Regularization

In the above steps, a *D*-dimensional random approximate function z(x) is used to approximate the unknown mapping function ϕ(x). In general, a large *D* can lead to small approximation error and good classification performance. However, an overlarge *D* may cause redundancy, which wastes storage space, and brings high computational complexity and high communication costs. There is a trade-off between the above two aspects, so we added a sparse regularization term. The regularization term pushes some dimensions of $w_{m}$ to 0, which means that these dimensions are redundant and can be discarded. When some dimensions of $w_{m}$ converge to 0, these dimensions do not need to be calculated, stored and transmitted.

The $l_{0}$-norm is typically used to measure sparsity. However, it is nonconvex, and $l_{0}$-norm-based problems are NP-hard. In practice, we can use the $l_{1}$-norm as a convex approximation of the $l_{0}$-norm. Introducing the $l_{1}$-norm into the objective function in (7), we obtain

(8) $$\begin{array}{cc} \min\;\; & \sum_{m=1}^{M}\left[\left(1-\alpha \right)\frac{1}{2}∥w_{m}{∥}^{2}+\alpha {∥w_{m}∥}_{1}\right]\\ \; & +C\sum_{m=1}^{M}\sum_{q=1}^{Q-1}\sum_{p=1}^{q}\sum_{i=1}^{N_{m}^{p}}L\left(b_{m,q}-w_{m}\cdot z\left(x_{m,i}^{p}\right)\right)\\ \; & +C\sum_{m=1}^{M}\sum_{q=1}^{Q-1}\sum_{p=q+1}^{Q}\sum_{i=1}^{N_{m}^{p}}L\left(w_{m}\cdot z\left(x_{m,i}^{p}\right)-b_{m,q}\right)\\ s.t.\;\; & w_{m}=w_{n},b_{m}=b_{n},\forall \left(m,n\right)\in E, \end{array}$$

where α∈[0,1] controls the proportion of the $l_{1}$-norm sparsity regularization term in the entire regularization term. A larger α can lead to a sparser solution of $w_{m}$. Therefore, since we set a relatively large *D* to ensure good performance, we could set a relatively large α to reduce redundancy.

We could view this problem from another perspective. If the last two terms in (8) are regarded to be the objective function, the first two terms combined together can be seen as a similar penalty to the elastic net penalty in [32], where α measures the weight of the $l_{1}$-norm penalty term.

After the above steps, we transformed Problem (3) into a convex optimization problem with consensus constraints (8).

### 4.5. Distributed SVOR Algorithm

In this subsection, we propose the dSVOR algorithm to solve Problem (8). First, we used the following notation for convenience

(9) $$\begin{array}{cc} J_{m}\left(\theta_{m}\right) & =\left(1-\alpha \right)\frac{1}{2}∥w_{m}{∥}^{2}+\alpha {∥w_{m}∥}_{1}+C\sum_{q=1}^{Q-1}\sum_{p=1}^{q}\sum_{i=1}^{N_{m}^{p}}L\left(b_{m,q}-w_{m}\cdot z\left(x_{m,i}^{p}\right)\right)\\ \; & \;+C\sum_{q=1}^{Q-1}\sum_{p=q+1}^{Q}\sum_{i=1}^{N_{m}^{p}}L\left(w_{m}\cdot z\left(x_{m,i}^{p}\right)-b_{m,q}\right), \end{array}$$

which is a convex function. The calculation of $J_{m}\left(\theta_{m}\right)$ does not need the data and estimated parameters from other nodes. Then, Problem (8) can be rewritten as follows:

(10) $$\begin{array}{cc} \min\;\; & J=\sum_{m=1}^{M}J_{m}\left(\theta_{m}\right)\\ s.t.\;\; & \theta_{m}=\theta_{n},\forall \left(m,n\right)\in E. \end{array}$$

To deal with consensus constraints $\theta_{m}=\theta_{n},\forall \left(m,n\right)\in E$, we adopted the penalty function method. The penalty function used in this paper is ${∥\theta_{m}-\theta_{n}∥}^{2}$, and the corresponding positive penalty coefficient is $\lambda_{mn}$. Then, the optimization problem becomes

(11) $$\begin{array}{c} \min\;\sum_{m=1}^{M}J_{m}\left(\theta_{m}\right)+\sum_{(m,n)\in E}\lambda_{mn}{∥\theta_{m}-\theta_{n}∥}^{2} \end{array}$$

The larger the $\lambda_{mn}$ is, the closer the solutions of Problems (11) and (10) are.

We then applied the subgradient method to optimize Problem (11). For the hinge loss function L(x)=max(1−x,0), we adopted the following subgradient:

(12) $$L^{\prime }\left(x\right)=\left\{\begin{array}{cccc} \; & -1, & x<1 & \\ \; & 0, & x\geq 1 \end{array}\right.,$$

and for the $l_{1}$-norm, we adopted

(13) $$sgn\left(x\right)=\left\{\begin{array}{cccc} \; & 1, & x>0 & \\ \; & -1, & x<0 & \\ \; & 0, & x=0 \end{array}\right..$$

At step k+1, the iterative equation is

(14) $$\begin{array}{cc} \theta_{m}^{k+1} & =\theta_{m}^{k}-\eta^{k}\nabla_{\theta_{m}}J_{m}\left(\theta_{m}^{k}\right)-2\eta^{k}\sum_{n\in N_{m}}\lambda_{mn}\left(\theta_{m}^{k}-\theta_{n}^{k}\right), \end{array}$$

where $\eta^{k}$ is the step size in step k+1, which is positive. The specific subgradients are

(15) $$\begin{array}{cc} \nabla_{w_{m}}J_{m}\left(\theta_{m}^{k}\right)\; & =\left(1-\alpha \right)w_{m}^{k}+\alpha sgn\left(w_{m}^{k}\right)\\ \; & \;-C\sum_{q=1}^{Q-1}\sum_{p=1}^{q}\sum_{i=1}^{N_{m}^{p}}L^{\prime }\left(b_{m,q}^{k}-w_{m}^{k}\cdot z\left(x_{m,i}^{p}\right)\right)z\left(x_{m,i}^{p}\right)\\ \; & \;+C\sum_{q=1}^{Q-1}\sum_{p=q+1}^{Q}\sum_{i=1}^{N_{m}^{p}}L^{\prime }\left(w_{m}^{k}\cdot z\left(x_{m,i}^{p}\right)-b_{m,q}^{k}\right)z\left(x_{m,i}^{p}\right), \end{array}$$

(16) $$\begin{array}{cc} \nabla_{b_{m,q}}J_{m}\left(\theta_{m}^{k}\right) & =C\sum_{p=1}^{q}\sum_{i=1}^{N_{m}^{p}}L^{\prime }\left(b_{m,q}^{k}-w_{m}^{k}\cdot z\left(x_{m,i}^{p}\right)\right)\\ \; & \;-C\sum_{p=q+1}^{Q}\sum_{i=1}^{N_{m}^{p}}L^{\prime }\left(w_{m}^{k}\cdot z\left(x_{m,i}^{p}\right)-b_{m,q}^{k}\right). \end{array}$$

In the subgradient method, in order to converge to the optimal solution, step size $\eta^{k}$ should satisfy [33]

(17) $$\sum_{k=0}^{+\infty }\eta^{k}=+\infty ,\;\mathrm{and}\;\sum_{k=0}^{+\infty }{\left(\eta^{k}\right)}^{2}<+\infty .$$

We can rearrange Iterative Equation (14) as follows.

(18) $$\begin{array}{cc} \theta_{m}^{k+1} & =\left(1-2\eta^{k}\sum_{n\in N_{m}}\lambda_{mn}\right)\theta_{m}^{k}+\sum_{n\in N_{m}}2\eta^{k}\lambda_{mn}\theta_{n}^{k}-\eta^{k}\nabla_{\theta_{m}}J_{m}\left(\theta_{m}^{k}\right). \end{array}$$

If we use the following notations for convenience

(19) $$\begin{array}{cc} \; & c_{mn}=2\eta^{k}\lambda_{mn},\;c_{mm}=1-\sum_{n\in N_{m}}2\eta^{k}\lambda_{mn}, \end{array}$$

the iterative equation can be rewritten as

(20) $$\begin{array}{c} \theta_{m}^{k+1}=\sum_{n\in N_{m}\cup \left\{m\right\}}c_{mn}\theta_{n}^{k}-\eta^{k}\nabla_{\theta_{m}}J_{m}\left(\theta_{m}^{k}\right). \end{array}$$

It can be divided into two steps, i.e., a combination step and an adaption step:

(21) $$\phi_{m}^{k}=\sum_{n\in N_{m}\cup \left\{m\right\}}c_{mn}\theta_{n}^{k},$$

(22) $$\theta_{m}^{k+1}=\phi_{m}^{k}-\eta^{k}\nabla_{\theta_{m}}J_{m}\left(\theta_{m}^{k}\right).$$

In Combination Step (21), node *m* combines the parameters estimated by its neighbors and itself to obtain an intermediate estimate $\phi_{m}^{k}$, where the combination coefficient of node *m* and its neighbor *n* is denoted as $c_{mn}$. In Adaption Step (22), node *m* uses the subgradient calculated by using only its own data to update $\theta_{m}$.

Combination coefficients ${\left\{c_{mn}\right\}}_{\forall (m,n)\in E}$ represent a cooperation rule among nodes. Equation (19) was not used to define $\left\{c_{mn}\right\}$ because $\lambda_{mn}$ was not defined in advance. In distributed algorithms, combination coefficients are generally determined by a certain cooperative protocol. In this study, we used the Metropolis rule [34]:

(23) $$c_{mn}=\left\{\begin{array}{cccc} \; & \frac{1}{\max(|N_{m}|,|N_{n}|)}, & n\in N_{m} & \\ \; & 1-\sum_{n\in N_{m}}c_{mn}, & m=n & \\ \; & 0, & \mathrm{otherwise} \end{array}\right.,$$

where $|N_{m}|$ denotes the degree of node *m*, and

(24) $$C1=1,1^{T}C=1^{T},$$

where C is an M×M matrix whose entries are defined by (23).

Equation (19) shows that $\lambda_{mn}=\frac{c_{mn}}{2\eta^{k}}$. Step size $\eta^{k}$ satisfies (17), where the latter implies that $\lim_{k\to \infty }\eta^{k}=0$. As k→∞, step size $\eta^{k}\to 0$ and penalty coefficient $\lambda_{mn}\to \infty$, which renders the solutions of Problems (11) and (10) nearly equal.

The whole processes of dSVOR are summarized in Algorithm 1.
**Algorithm 1** Distributed SVOR algorithm**Initialization:** initialize hinge loss function weight *C*, sparsity regularization weight α, random approximate dimension *D*, and total iteration number *T*. Each node *m* initializes $\theta_{m}=\left\{w_{m},b_{m}\right\}$.**for**k=1:T   **for** m=1:M      **Communication Step:** communicate parameters $\theta_{m}$ with neighbors $n\in N_{m}$.   **end for**   **for** m=1:M      **Combination Step:** compute intermediate estimate $\phi_{m}^{k}$ via (21).      **Computation Step:** Compute the subgradients $\nabla_{w_{m}}J_{m}\left(\theta_{m}^{k}\right)$, $\nabla_{b_{m,q}}J_{m}\left(\theta_{m}^{k}\right)$ via (15) and (16);      **Adaption Step:** update $\theta_{m}^{k+1}$ via (22).   **end for****end for**

**Remark** **1.** *In the above problems, ϕ(·) is a nonlinear mapping function that maps input x into a RKHS for classification, and input x is the original data or extracted features. In general, function ϕ(·) can also be regarded to be a generalized feature mapping function that extracts features of x, and maps x into a feature space for classification. Thus, it can also use an artificial neural network with learnable parameters. However, that may destroy the convexity of the problem, so that it is no longer guaranteed to converge to the global optimum.*

### 4.6. Theoretical Analysis

In this subsection, we theoretically analyze the consensus and convergence of dSVOR.

We first introduce a reasonable assumption that is needed in analysis. According to [34], when the graph is not bipartite, this assumption can be guaranteed.

**Assumption** **1.** *Spectral radius $\rho (C-\frac{1}{M}11^{T})<1$, where C is the combination coefficient matrix set as in Equation (23).*

Then, we give two theorems about consensus and convergence each.

**Theorem** **1** (Consensus). *If Assumption 1 holds, and step size $\eta^{k}$ satisfies Condition (17), then $\lim_{k\to \infty }∥\theta_{m}^{k}-{\bar{\theta }}^{k}∥=0,\forall m$, where ${\bar{\theta }}^{k}=\frac{1}{M}\sum_{m=1}^{M}\theta_{m}^{k}$.*

**Theorem** **2** (Convergence). If Assumption 1 holds, and step size $\eta^{k}$ satisfies Condition (17), then $\lim_{k\to \infty }\sum_{m=1}^{M}J_{m}\left(\theta_{m}^{k}\right)=J^{*}$, where $J^{*}=\min J$.

For the proof, see Appendix A and Appendix B for details.

## 5. Experiments

In this section, we carry out experiments on synthetic data and a real-world example to demonstrate the performance of the proposed dSVOR algorithm.

We implemented the following algorithms for comparison:

1. proposed dSVOR algorithm (dSVOR);
2. centralized SVOR (cSVOR), which relies on all the data available in a central node;
3. distributed SVOR with a noncooperative strategy (ncSVOR). In ncSVOR, each node uses only its own data to train a model without any information exchanged with other nodes.

All the algorithms were implemented using the PyTorch framework [35].

There are three points to emphasize:

1. The centralized method needs data in a central node. For comparison, we artificially collected all the data distributed in the nodes of the network together to render it applicable, which is impractical in reality.
2. In cSVOR [11,12], problems were solved by the SMO algorithm instead of subgradient-based algorithms, so we only display its final results.
3. The distributed algorithms were subject to additional constraints, so a distributed algorithm is generally satisfactory if it can achieve comparable performance to the corresponding centralized algorithm.

In this study, we used the prediction accuracy (ACC) and mean absolute error (MAE) on the testing set as the performance evaluation metrics. ACC is a commonly used metric in classification problems, but it does not consider the ordered information of the labels. MAE is the mean absolute deviation of the predicted rank from the true one, which is commonly used in ordinal regression. Using a function O(·) to denote the position of a certain label in the ordinal scale, i.e., $O(C_{q})=q,q=1,\ldots ,Q$, we have

(25) $$MAE=\frac{1}{N}\sum_{i=1}^{N}\left|O\left(y_{i}\right)-O\left({\hat{y}}_{i}\right)\right|\in \left[0,Q-1\right].$$

The performance of distributed algorithms (dSVOR and ncSVOR) is defined as the mean performance of models obtained by each node. The distributed algorithms ran on a randomly generated connected network that consisted of 20 nodes. For fair comparison, on a certain dataset, all implemented algorithms used the same parameters. All the results were obtained by averaging the results of 10 independent experiments.

### 5.1. Synthetic Data

**Figure 2:**
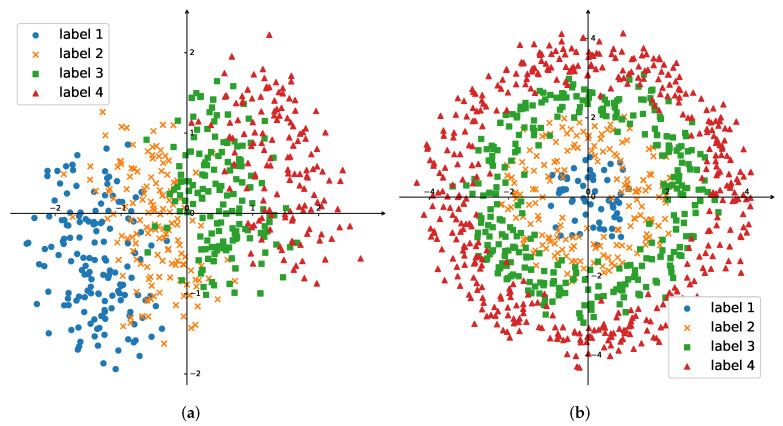
Data visualization of the (**a**) first and (**b**) second synthetic datasets.

In this subsection, we evaluate the performance of all algorithms on two synthetic datasets. On the first dataset, samples could be separated by a set of parallel straight lines if ignoring noises, and samples of the second dataset could be separated by a set of concentric circles. Figure 2a,b show some samples of these two datasets from one of the 10 independent experiments. Both datasets had 1200 samples: 1000 were used as the training set, and the others were the testing set. The training samples were randomly assigned to 20 nodes to simulate the situation where the data were collected and stored by these nodes in a distributed manner.

These two synthetic datasets were generated with the following methods. For the first dataset, we generated 1200 samples with uniform distribution from a rectangular area $x_{1min}\leq x_{1}\leq x_{1max},x_{2min}\leq x_{2}\leq x_{2max}$. We then used three straight lines ${\left\{x_{1}=b_{i}\right\}}_{i=1,2,3}$ to divide this area into 4 parts for 4 classes. The data labels were determined by their locations. Then, Gaussian noise with 0 mean and $\sigma_{1}$ standard deviation was added to each dimension of input vector $x={\left[x_{1}\;x_{2}\right]}^{T}$. After that, these samples were rotated around the origin with β. Without loss of generality, in the experiments, these parameters were set as follows:

$$\begin{array}{c} x_{1min}=-2,x_{1max}=2,x_{2min}=-1,x_{2max}=1,\\ b_{1}=-1,b_{2}=0,b_{3}=1,\sigma_{1}=0.5,\beta =\frac{\pi }{8}. \end{array}$$

For the second dataset, we generated 1200 samples with uniform distribution from a circle $x_{1}^{2}+x_{2}^{2}<R^{2}$, which could be divided into four parts by three concentric circles ${\left\{x_{1}^{2}+x_{2}^{2}=R_{i}^{2}\right\}}_{i=1,2,3}$. The data labels were determined by their locations. Then, Gaussian noise with 0 mean and $\sigma_{2}$ standard deviation was added to each dimension of input vector $x={\left[x_{1}\;x_{2}\right]}^{T}$. Without loss of generality, the parameters were set to be R=4, $R_{1}=1$, $R_{2}=2$, $R_{3}=3$, $\sigma_{2}=0.2$.

On the first dataset, we used a linear kernel function. In all methods, positive constant *C* was set to be 1000/N, where *N* is the number of samples of all nodes. Because the feature space was only 2-dimensional, the sparse regularization term in our method was not necessary. Thus, we set the coefficient of sparse regularization term α=0. In the distributed algorithm, we used the following diminishing step size:

(26) $$\eta^{k}=\frac{\eta^{0}}{1+\tau k},$$

which satisfied Condition (17). In (26), parameter $\eta^{0}$ determines the initial step size, and τ determines the decreasing rate of the diminishing step size. We empirically set $\eta^{0}=0.1$ and τ=0.01 in the following experiments.

**Figure 3:**
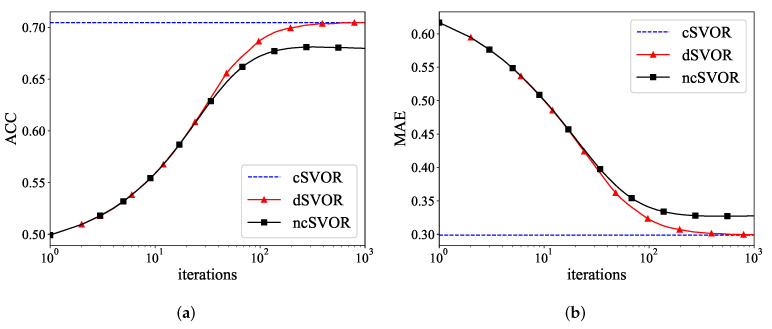
(**a**) ACC and (**b**) MAE curves of different algorithms on the first synthetic dataset.

Figure 3a,b show the ACC and MAE curves of different algorithms on the first synthetic dataset. As time increased, the MAE of our dSVOR algorithm decreased, and the ACC increased significantly. After about 500 iterations, the dSVOR algorithm converged to a value that was almost the same as that of cSVOR, while the result of ncSVOR was still some distance away from them. This means that it was not enough for a single node to train a model with good performance using its own data. The proposed dSVOR algorithm, which uses the local data of each node and the parameter estimates from neighbor nodes, could achieve a similar performance to that of the corresponding centralized method.

**Figure 4:**
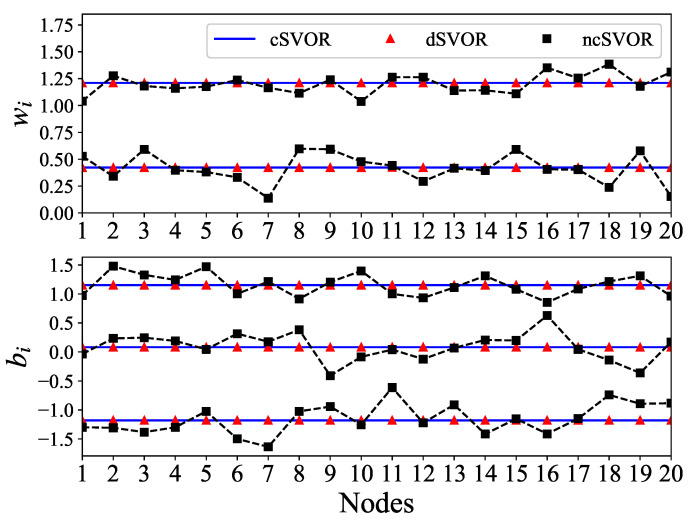
Final estimated parameters of different methods.

Figure 4 gives the parameters of each node estimated by different algorithms. In the ncSVOR algorithm, the estimated parameters obtained by different nodes were quite different. Thus, the model obtained by each node with its own data was quite different from the model trained using all the data. In contrast, the estimated parameters of different nodes in dSVOR were almost the same as the parameters in cSVOR. This illustrates the consensus of the proposed dSVOR algorithm. Because we used a linear kernel function here, optimal direction w in the centralized method had an explicit expression that allowed for us to compare it with the estimates of the distributed algorithms. In the following experiments using nonlinear kernel functions, we do not give the results about consensus.

On the second dataset, we used a Gaussian kernel function. The kernel size was set to be $\sigma =\frac{1}{K}$ after Z-score normalization, where *K* is the dimension of input space. In all methods, positive constant *C* was set to be 1000/N. As analyzed before, in our method, we set a relatively large *D* and a relatively large *a*, D=200, α=0.9. α was not set to 1 because we wanted to use the strong convexity of the $l_{2}$-norm regularization term to increase the convexity of the objective function, which is theoretically beneficial to the optimization of the problem. The learning rate parameters were still set to be $\eta^{0}=0.1$ and τ=0.01.

**Figure 5:**
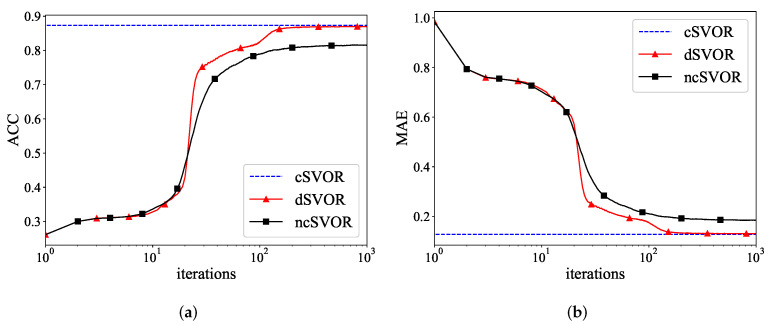
(**a**) ACC and (**b**) MAE curves of different algorithms on the second synthetic dataset.

Figure 5a,b show the ACC and MAE curves of different algorithms on the second synthetic dataset. The proposed dSVOR algorithm was able to obtain almost the same result as that of the centralized method, while ncSVOR could not.

**Figure 6:**
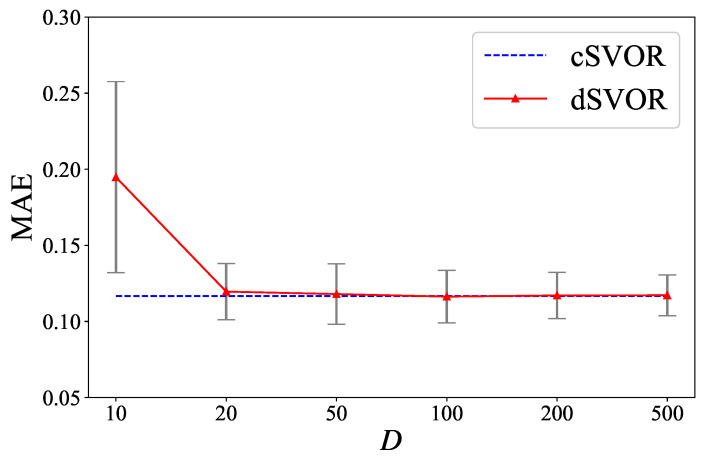
MAEs of dSVOR on the second synthetic dataset for different *D* when α is fixed as 0.9.

**Figure 7:**
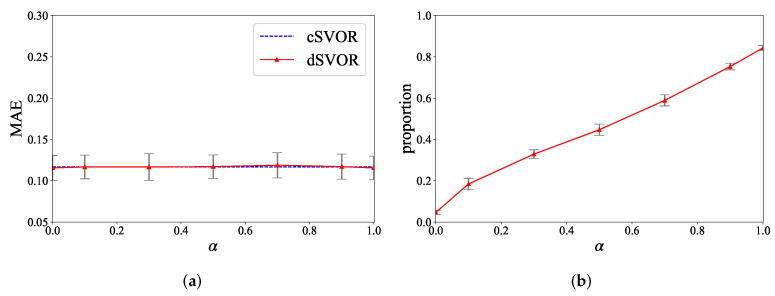
Results of dSVOR on the second synthetic dataset for different α when *D* is fixed as 200. (**a**) MAEs; (**b**) proportions of dimensions of $w_{m}$ that are equal to 0.

We also conducted experiments under different hyperparameters *D* and α to show the parameter sensitivity of dSVOR. Figure 6 gives the MAEs of dSVOR for different *D* when α was fixed as 0.9. As *D* increased, the performance of dSVOR gradually improved and was eventually almost the same as that of the centralized method. With a relatively large approximation dimension D≥100, dSVOR could always obtain a similar MAE to that of cSVOR. However, as mentioned before, an overlarge *D* may cause redundancy. So, when using a large *D* to ensure good performance, it is better to use the sparse regularization term to reduce the redundancy. Figure 7a,b gives the MAEs of dSVOR and the proportions of dimensions of $w_{m}$ that were equal to 0 for different α when *D* is fixed as 200. The MAE was stable under different α, but the sparsity of $w_{m}$ was greatly affected by α. A small α led to a dense $w_{m}$, which caused a lot of redundancy. A large α could bring a sparse $w_{m}$, where the dimensions that converged to 0 could no longer be stored, calculated, and transmitted after converging to 0, thus saving storage, computation, and communication resources.

### 5.2. A Real-World Example

We now take the distributed fault severity diagnosis of rolling element bearings as a real-world example to illustrate the effectiveness of dSVOR.

Rolling element bearings are widely used in factory equipments. The fault severity diagnosis of bearings is a crucial task to ensure reliability in industrial processes. In recent years, data-driven methods have been widely used to identify faults and their severity [36]. To achieve good performance, these data-driven methods usually require a lot of data. However, due to the rarity of faults, a single sensor can only collect very few fault data, and the faults encountered by each factory may also be different. Thus, data from many sensors in many factories are needed to train a proper model. Sometimes, factories may not want to leak the data about their equipments, so it is not allowed to transmit the data to others. The centralized methods which need all the data available in a central node become inapplicable. The distributed methods become a better choice. Taking into account the ordinal information in the fault severity, it is suitable to apply the proposed dSVOR algorithm.

In this study, we used the rolling element bearings data provided by the Case Western Reserve University (CWRU) [37] for experiments. CWRU data were the vibration signals of drive end and fan end bearings collected by sensors at 12,000 and 48,000 samples/s under four different loads of 0–3 hp. There are three types of faults: outer race (OR), inner race (IR), and ball (B) faults, and each type has at most four severity levels (fault width: 0.18, 0.36, 0.53, 0.71 mm). In the experiments, we used drive end bearing data collected at 12,000 samples/s, and performed 4-level fault severity diagnosis in a total of 12 situations (3 different fault types and 4 different loads).

We adopted the feature based on permutation entropy (PE) proposed in [38] as the input x. For one datum, we intercepted a sequence of length 2400 from vibration signal data. This sequence was decomposed into a series of intrinsic mode functions (IMFs) by ensemble empirical mode decomposition (EEMD) with 100 ensembles and 0.2 noise amplitude to catch information on multiple time scales. Then, the PE values of the first 5 IMFs are calculated as the input feature of this piece of data.

For each fault severity level, we randomly took 300 training samples and 200 testing samples, and the samples in the testing set were different from those in the training set. For 4-level fault level diagnosis, there were a total of 1200 training samples and 800 testing samples. These training samples were randomly assigned to 20 nodes to simulate the situation where the data were collected and stored by these nodes in a distributed manner.

In the experiments, we used a Gaussian kernel function with kernel size $\sigma =\frac{1}{K}$ after Z-score normalization. In all methods, positive constant *C* was set to be 10,000/N. In our method, we still set a relatively large hyperparameter *D* and α, D=200, α=0.9. The other parameters used the same settings as before, i.e., $\eta^{0}=0.1$ and τ=0.01.

Table 1 shows the experimental results where the value was the mean ± standard deviation of 10 independent experiments. The performance of ncSVOR was worse than that of cSVOR because each node only had part of the training samples that were not enough to represent the entire training set to train a proper model. Compared to ncSVOR, the proposed dSVOR algorithm could achieve similar results to those of cSVOR. In dSVOR, each node can only use the data of its own and exchange some estimated parameters with neighbor nodes. It was satisfactory to be able to achieve performance close to that of the centralized method that uses all the data from all nodes.

**Figure 8:**
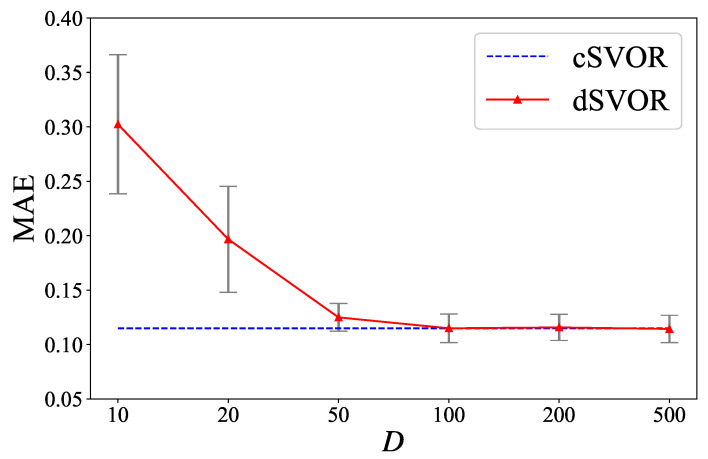
MAEs of dSVOR on the CWRU dataset of IR fault type and 0 hp load for different *D* when α is fixed as 0.9.

**Figure 9:**
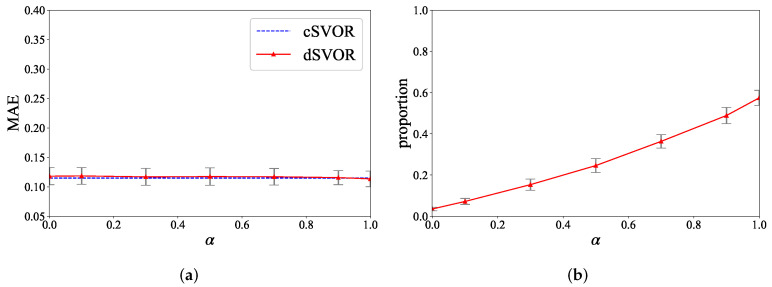
The results of dSVOR on the CWRU dataset of IR fault type and 0 hp load for different α when *D* is fixed as 200 (**a**) the MAEs (**b**) the proportions of dimensions of $w_{m}$ that are equal to 0.

Taking the dataset of the IR fault type and 0 hp load as examples, we also show the results of dSVOR under different hyperparameters *D* and α in Figure 8 and Figure 9. Figure 8 shows that, with a relatively large random approximation dimension D≥100, dSVOR could obtain a similar MAE to that of cSVOR, which illustrates the effectiveness of the random approximation. Figure 9 shows that a relatively large α can lead to a sparse $w_{m}$ without affecting the MAE performance, thus effectively reducing redundancy.

## 6. Conclusions

When data are distributedly collected and stored by multiple nodes, and are difficult to transmit to a central node, existing centralized ordinal regression methods become inapplicable. To this end, in order to handle the ordinal regression problem in distribution scenarios, we extended the SVORIM to a distributed version, and derived a distributed SVOR (dSVOR) algorithm. In dSVOR, each node combines the parameters estimated by its neighbors and performs local calculations using only its own data. After convergence, each node can obtain a model whose performance is close to that obtained by the centralized method relying on all the data available in a central node. Theoretically, we analyzed the consensus and the convergence of dSVOR. Practically, we carried out experiments on synthetic data and a real-world example to illustrate its effectiveness.

In our future work, we intend to consider how to automatically determine the proper parameters in dSVOR, e.g., introducing multi-kernel learning to automatically find suitable parameters of random approximate. We also aim to design adaptive strategies for adjusting combination coefficients.

## Figures and Tables

**Table 1:** ACCs and MAEs of different algorithms in a real-world example (mean ± std).

Fault Type	Load	cSVOR		ncSVOR		dSVOR	
		ACC	MAE	ACC	MAE	ACC	MAE
>OR	0	0.9585 ± 0.0057	0.0415 ± 0.0057	0.7977 ± 0.0211	0.2069 ± 0.0230	0.9553 ± 0.0064	0.0447 ± 0.0064
	1	0.9317 ± 0.0147	0.0683 ± 0.0147	0.7376 ± 0.0228	0.2726 ± 0.0264	0.9278 ± 0.0136	0.0727 ± 0.0138
	2	0.9547 ± 0.0091	0.0457 ± 0.0096	0.7901 ± 0.0136	0.2172 ± 0.0153	0.9517 ± 0.0094	0.0492 ± 0.0099
	3	0.9253 ± 0.0099	0.0747 ± 0.0099	0.7599 ± 0.0158	0.2489 ± 0.0173	0.9243 ± 0.0095	0.0758 ± 0.0096
>IR	0	0.8853 ± 0.0133	0.1149 ± 0.0133	0.7472 ± 0.0087	0.2589 ± 0.0091	0.8844 ± 0.0120	0.1157 ± 0.0120
	1	0.8624 ± 0.0112	0.1376 ± 0.0112	0.7288 ± 0.0103	0.2781 ± 0.0110	0.8556 ± 0.0137	0.1444 ± 0.0137
	2	0.8435 ± 0.0109	0.1565 ± 0.0109	0.7071 ± 0.0116	0.3000 ± 0.0133	0.8391 ± 0.0113	0.1611 ± 0.0113
	3	0.8726 ± 0.0095	0.1291 ± 0.0091	0.7238 ± 0.0110	0.2918 ± 0.0122	0.8632 ± 0.0094	0.1392 ± 0.0091
B	0	0.7768 ± 0.0110	0.2586 ± 0.0129	0.5440 ± 0.0184	0.5975 ± 0.0311	0.7594 ± 0.0221	0.2771 ± 0.0245
	1	0.7836 ± 0.0105	0.2419 ± 0.0099	0.5770 ± 0.0124	0.5284 ± 0.0195	0.7710 ± 0.0067	0.2540 ± 0.0106
	2	0.8256 ± 0.0088	0.1886 ± 0.0088	0.5820 ± 0.0156	0.5341 ± 0.0264	0.8177 ± 0.0147	0.1980 ± 0.0150
	3	0.8627 ± 0.0167	0.1541 ± 0.0193	0.6345 ± 0.0138	0.4648 ± 0.0253	0.8485 ± 0.0169	0.1710 ± 0.0204

## Data Availability

Not applicable.

## Appendix A. Proof of Theorem 1

**Proof.** For convenience, we use the following notation:

(A1) $$\begin{array}{c} \theta^{k}={\left[\theta_{1}^{k},\ldots ,\theta_{M}^{k}\right]}^{T},\;G_{\theta }^{k}={\left[\nabla_{\theta_{1}}J_{1}\left(\theta_{1}^{k}\right),\ldots ,\nabla_{\theta_{M}}J_{M}\left(\theta_{M}^{k}\right)\right]}^{T}. \end{array}$$

Then, Iterative Equation (20) can be written as follows.

(A2) $$\theta^{k+1}=C\theta^{k}-\eta^{k}G_{\theta }^{k}.$$

Considering ${\bar{\theta }}^{k}=\frac{1}{M}1^{T}\theta^{k}$, the proof of $\lim_{k\to \infty }∥\theta_{m}^{k}-{\bar{\theta }}^{k}∥=0,\forall m$ can be done by proving $\lim_{k\to \infty }∥\theta^{k}-\frac{1}{M}11^{T}\theta^{k}∥=0$. We first construct

(A3) $$\begin{array}{cc} \theta^{k+1}-\frac{1}{M}11^{T}\theta^{k+1}\; & =\left(I-\frac{1}{M}11^{T}\right)\theta^{k+1}=\left(I-\frac{1}{M}11^{T}\right)\left(C\theta^{k}-\eta^{k}G_{\theta }^{k}\right)\\ \; & =\left(C-\frac{1}{M}11^{T}\right)\theta^{k}-\left(I-\frac{1}{M}11^{T}\right)\eta^{k}G_{\theta }^{k}. \end{array}$$

Notice that

(A4) $$C\frac{1}{M}11^{T}=\frac{1}{M}11^{T}=\frac{1}{M}11^{T}\frac{1}{M}11^{T}.$$

We have

(A5) $$\begin{array}{cc} \theta^{k+1}-\frac{1}{M}11^{T}\theta^{k+1} & =\left(C-\frac{1}{M}11^{T}\right)\theta^{k}-\left(I-\frac{1}{M}11^{T}\right)\eta^{k}G_{\theta }^{k}\\ \; & \;-C\frac{1}{M}11^{T}\theta^{k}+\frac{1}{M}11^{T}\frac{1}{M}11^{T}\theta^{k}\\ \; & =\left(C-\frac{1}{M}11^{T}\right)\left(\theta^{k}-\frac{1}{M}11^{T}\theta^{k}\right)-\left(I-\frac{1}{M}11^{T}\right)\eta^{k}G_{\theta }^{k}. \end{array}$$

For convenience, we use notation $\Delta \theta^{k}=\theta^{k}-\frac{1}{M}11^{T}\theta^{k}$, then

(A6) $$\begin{array}{c} \Delta \theta^{k+1}=\left(C-\frac{1}{M}11^{T}\right)\Delta \theta^{k}-\left(I-\frac{1}{M}11^{T}\right)\eta^{k}G_{\theta }^{k}. \end{array}$$

We then prove $\lim_{k\to \infty }∥\Delta \theta^{k}∥=0$. Taking the $l_{2}$-norm on both sides of the above equation, we have

(A7) $$\begin{array}{cc} ∥\Delta \theta^{k+1}∥\; & \leq ∥C-\frac{1}{M}11^{T}∥∥\Delta \theta^{k}∥+\eta^{k}∥I-\frac{1}{M}11^{T}∥∥G_{\theta }^{k}∥=\rho ∥\Delta \theta^{k}∥+\eta^{k}c∥G_{\theta }^{k}∥, \end{array}$$

where $c=∥I-\frac{1}{M}11^{T}∥$ is a positive constant, and ρ denotes the spectral norm of $C-\frac{1}{M}11^{T}$, which <1 according to Assumption A1. Since $J_{m}\left(\theta_{m}\right)$ is Lipschitz continuous, $G_{\theta }^{k}$ is bounded, so there exists a positive constant *L* satisfying $∥G_{\theta }^{k}∥\leq L$. Thus,

(A8) $$∥\Delta \theta^{k+1}∥\leq \rho ∥\Delta \theta^{k}∥+\eta^{k}cL.$$

Now we prove that $\lim_{k\to \infty }∥\Delta \theta^{k}∥=0$. To achieve this, we constructed an auxiliary variable $u^{k}$ that satisfied

(A9) $$u^{k+1}=\rho u^{k}+\eta^{k}cL,$$

and $u^{0}=∥\Delta \theta^{0}∥\geq 0$. If $u^{k}\geq ∥\Delta \theta^{k}∥\geq 0$, then

(A10) $$u^{k+1}=\rho u^{k}+\eta^{k}cL\geq \rho ∥\Delta \theta^{k}∥+\eta^{k}cL\geq ∥\Delta \theta^{k+1}∥.$$

So $u^{k}\geq ∥\Delta \theta^{k}∥\geq 0$ for all k≥0. With ρ<1 and $\lim_{k\to \infty }\eta^{k}=0$, we have $\lim_{k\to \infty }u^{k}=0$. Then

(A11) $$0\leq \lim_{k\to \infty }∥\Delta \theta^{k}∥\leq \lim_{k\to \infty }u^{k}=0.$$

So we have

(A12) $$\lim_{k\to \infty }∥\theta^{k}-\frac{1}{M}11^{T}\theta^{k}∥=\lim_{k\to \infty }∥\Delta \theta^{k}∥=0.$$

The proof of Theorem 1 is completed. □

## Appendix B. Proof of Theorem 2

**Proof.** From Equation (20), we can obtain

(A13) $$\begin{array}{cc} \sum_{m=1}^{M}\theta_{m}^{k+1} & =\sum_{m=1}^{M}\sum_{n\in N_{m}\cup \left\{m\right\}}c_{mn}\theta_{n}^{k}-\sum_{m=1}^{M}\eta^{k}\nabla_{\theta_{m}}J_{m}\left(\theta_{m}^{k}\right)\\ \; & =\sum_{m=1}^{M}\theta_{m}^{k}-\sum_{m=1}^{M}\eta^{k}\nabla_{\theta_{m}}J_{m}\left(\theta_{m}^{k}\right). \end{array}$$

From Theorem 1, we have $\lim_{k\to \infty }∥\theta_{m}^{k}-{\bar{\theta }}^{k}∥=0$. So, for a sufficiently large $k=k_{1}$, the above equation can be written as follows:

(A14) $${\bar{\theta }}^{k_{1}+1}={\bar{\theta }}^{k_{1}}-\frac{\eta^{k_{1}}}{M}\sum_{m=1}^{M}\nabla_{\theta_{m}}J_{m}\left({\bar{\theta }}^{k_{1}}\right),$$

where $\nabla_{\theta_{m}}J_{m}\left({\bar{\theta }}^{k_{1}}\right)$ denotes the subgradient of $J_{m}\left(\theta_{m}\right)$ with respect to $\theta_{m}$ when $\theta_{m}={\bar{\theta }}^{k_{1}}$. Supposing $\theta^{*}=\arg\min J$, we have

(A15) $$\begin{array}{cc} ∥{\bar{\theta }}^{k_{1}+1}-\theta^{*}{∥}^{2} & =∥{\bar{\theta }}^{k_{1}}-\frac{\eta^{k_{1}}}{M}\sum_{m=1}^{M}\nabla_{\theta_{m}}J_{m}\left({\bar{\theta }}^{k_{1}}\right)-\theta^{*}{∥}^{2}\\ \; & =∥{\bar{\theta }}^{k_{1}}-\theta^{*}{∥}^{2}+{∥\frac{\eta^{k_{1}}}{M}\sum_{m=1}^{M}\nabla_{\theta_{m}}J_{m}\left({\bar{\theta }}^{k_{1}}\right)∥}^{2}\\ \; & \;-2\frac{\eta^{k_{1}}}{M}\sum_{m=1}^{M}\nabla_{\theta_{m}}J_{m}{\left({\bar{\theta }}^{k_{1}}\right)}^{T}\left({\bar{\theta }}^{k_{1}}-\theta^{*}\right). \end{array}$$

Since $J_{m}\left(\theta_{m}\right)$ is Lipschitz continuous for all *m*, there exists a positive constant *L* satisfying $∥\frac{1}{M}\sum_{m=1}^{M}\nabla_{\theta_{m}}J_{m}\left(\theta_{m}\right){∥}^{2}\leq L^{2}$. Thus,

(A16) $$\begin{array}{cc} ∥{\bar{\theta }}^{k_{1}+1}-\theta^{*}{∥}^{2}\; & \leq ∥{\bar{\theta }}^{k_{1}}-\theta^{*}{∥}^{2}+{\left(\eta^{k_{1}}\right)}^{2}L^{2}-2\frac{\eta^{k_{1}}}{M}\sum_{m=1}^{M}\nabla_{\theta_{m}}J_{m}{\left({\bar{\theta }}^{k_{1}}\right)}^{T}\left({\bar{\theta }}^{k_{1}}-\theta^{*}\right). \end{array}$$

Because $J_{m}\left(\theta_{m}\right)$ is convex, we have

(A17) $$\begin{array}{c} \nabla_{\theta_{m}}J_{m}{\left({\bar{\theta }}^{k_{1}}\right)}^{T}\left({\bar{\theta }}^{k_{1}}-\theta^{*}\right)\geq J_{m}\left({\bar{\theta }}^{k_{1}}\right)-J_{m}\left(\theta^{*}\right). \end{array}$$

Then

(A18) $$\begin{array}{cc} ∥{\bar{\theta }}^{k_{1}+1}-\theta^{*}{∥}^{2}\; & \leq ∥{\bar{\theta }}^{k_{1}}-\theta^{*}{∥}^{2}+{\left(\eta^{k_{1}}\right)}^{2}L^{2}-2\frac{\eta^{k_{1}}}{M}\sum_{m=1}^{M}\left(J_{m}\left({\bar{\theta }}^{k_{1}}\right)-J_{m}\left(\theta^{*}\right)\right)\\ \; & =∥{\bar{\theta }}^{k_{1}}-\theta^{*}{∥}^{2}+{\left(\eta^{k_{1}}\right)}^{2}L^{2}-2\frac{\eta^{k_{1}}}{M}\left(J\left({\bar{\theta }}^{k_{1}}\right)-J^{*}\right). \end{array}$$

If $\lim_{k\to \infty }\sum_{m=1}^{M}J_{m}\left(\theta_{m}^{k}\right)\neq J^{*}$, $\exists \varepsilon >0,k_{2}>0$, $\forall k\geq k_{2},J\left({\bar{\theta }}^{k_{2}}\right)-J^{*}>\varepsilon$. Let $k_{3}=\max\left\{k_{1},k_{2}\right\}$. Then

(A19) $$\begin{array}{cc} ∥{\bar{\theta }}^{k_{3}+1}-\theta^{*}{∥}^{2}\; & <∥{\bar{\theta }}^{k_{3}}-\theta^{*}{∥}^{2}+{\left(\eta^{k_{3}}\right)}^{2}L^{2}-2\frac{\eta^{k_{3}}}{M}\varepsilon . \end{array}$$

Taking the summation of both sides of the above equation over $k=k_{3},\ldots ,k_{3}+k^{*}$, we obtain

(A20) $$\begin{array}{cc} ∥{\bar{\theta }}^{k_{3}+k^{*}}-\theta^{*}{∥}^{2}\; & <∥{\bar{\theta }}^{k_{3}}-\theta^{*}{∥}^{2}+\sum_{k=k_{3}}^{k_{3}+k^{*}}{\left(\eta^{k}\right)}^{2}L^{2}-\sum_{k=k_{3}}^{k_{3}+k^{*}}2\frac{\eta^{k}}{M}\varepsilon . \end{array}$$

Since $∥{\bar{\theta }}^{k_{3}+k^{*}}-\theta^{*}{∥}^{2}\geq 0$, we have

(A21) $$\begin{array}{c} ∥{\bar{\theta }}^{k_{3}}-\theta^{*}{∥}^{2}+\sum_{k=k_{3}}^{k_{3}+k^{*}}{\left(\eta^{k}\right)}^{2}L^{2}>\sum_{k=k_{3}}^{k_{3}+k^{*}}2\frac{\eta^{k}}{M}\varepsilon . \end{array}$$

Thus,

(A22) $$\begin{array}{c} \frac{∥{\bar{\theta }}^{k_{3}}-\theta^{*}{∥}^{2}+L^{2}\sum_{k=k_{3}}^{k_{3}+k^{*}}{\left(\eta^{k}\right)}^{2}}{\frac{2}{M}\sum_{k=k_{3}}^{k_{3}+k^{*}}\eta^{k}}>\varepsilon . \end{array}$$

Since $\sum_{k=0}^{+\infty }\eta^{k}=+\infty$ and $\sum_{k=0}^{+\infty }{\left(\eta^{k}\right)}^{2}<+\infty$,

(A23) $$\begin{array}{c} \lim_{k^{*}\to \infty }\frac{∥{\bar{\theta }}^{k_{3}}-\theta^{*}{∥}^{2}+L^{2}\sum_{k=k_{3}}^{k_{3}+k^{*}}{\left(\eta^{k}\right)}^{2}}{\frac{2}{M}\sum_{k=k_{3}}^{k_{3}+k^{*}}\eta^{k}}=0, \end{array}$$

which conflicts with Equation (A22). Thus,

(A24) $$\lim_{k\to \infty }\sum_{m=1}^{M}J_{m}\left(\theta_{m}^{k}\right)=J^{*}.$$

The proof of Theorem 2 is completed. □
